# Seroprevalence and molecular detection of *toxoplasma gondii* in sheep and pigs in Xinjiang Uygur autonomous region, China

**DOI:** 10.1016/j.fawpar.2026.e00340

**Published:** 2026-05-11

**Authors:** Wen-Ge Liu, Jia Huang, Maihemudaimu Maihemuti, Wang Fu, Hang-Yu Meng, Xiao-Pei Xu

**Affiliations:** a Department of Human Parasitology, School of Basic Medical Sciences, Xinjiang Medical University, Ürümqi, Xinjiang, China; b Institute of Pathogenic Biological Detection, Xinjiang Center for Disease Control and Prevention, Ürümqi 830000, Xinjiang/Uygur Autonomous Region, China; c Department of Human Anatomy, School of Basic Medical Sciences, Xinjiang Medical University, Ürümqi, Xinjiang, China

**Keywords:** Toxoplasma gondii, Seroprevalence, Food safety, Genotyping, Sheep, Pig

## Abstract

*Toxoplasma gondii* is a globally distributed, foodborne zoonotic protozoan parasite, yet systematic epidemiological data on its prevalence in local livestock remain limited in the Xinjiang Uygur Autonomous Region, China. This study determined the seroprevalence of *T. gondii* in sheep and pigs across Xinjiang, From April 2024 to July 2025, 1011 sheep and 700 pig serum samples were collected from farms in Northern, Southern, and Eastern Xinjiang. Antibodies were detected using indirect hemagglutination test (IHAT). Muscle tissue samples (300 sheep, 300 pigs) from retail outlets were analyzed by nested PCR targeting the B1 gene, and positive samples were genotyped using multilocus PCR-RFLP (Mn-PCR-RFLP) at 10 genetic markers. The overall seroprevalence was 11.8% (95% CI: 9.8–13.8) in sheep and 17.6% (95% CI: 14.8–20.4) in pigs. Significant risk factors for sheep included Southern Xinjiang region (OR = 2.22, *P* = 0.032), presence of cats (OR = 2.19, *P* = 0.001), extensive grazing (OR = 2.23, *P* = 0.002), and female sex (OR = 2.79, *P* = 0.003). For pigs, significant factors were presence of cats (OR = 1.81, *P* = 0.042) and age 2–3 years (OR = 2.58, *P* = 0.018). Tissue cyst DNA was detected in 8.7% (26/300) of sheep and 13.3% (40/300) of pigs. ToxoDB#9 and #10 were the predominant genotypes among successfully typed isolates (4/6 sheep and 6/8 pigs), with others showing mixed or alternative genotypes. These findings reveal widespread *T. gondii* infection in Xinjiang livestock, highlighting the need for improved farm biosecurity and public health education regarding safe meat consumption practices.

## Highlights

• The systematic investigation of *T. gondii* in Xinjiang reveals 11.8% seroprevalence in sheep and 17.6% in pigs, with tissue cysts detected in retail meat and ToxoDB #9/#10 as dominant genotypes.
• Grazing systems, feline presence, and Southern Xinjiang region are key risk factors; notably, no age-related trends indicate persistent environmental contamination across all age groups.
• Results highlight urgent food safety risks linked to local dietary practices (unpasteurized milk and undercooked meat), emphasizing the need for enhanced farm biosecurity and consumer education.

## 1 Introduction

*Toxoplasma gondii* is a globally distributed foodborne apicomplexan protozoan capable of infecting virtually all warm-blooded animals. Due to its substantial disease burden—including congenital toxoplasmosis, ocular lesions, and life-threatening encephalitis in immunocompromised individuals—the Food and Agriculture Organization (FAO) and World Health Organization (WHO) jointly rank it as the fourth most important foodborne parasite worldwide (Smith et al., 2021; Dubey, 2021; Elsheikha et al., 2020). In China, where meat consumption has risen dramatically with economic growth, the detection of *T. gondii* in food-producing animals has raised serious consumer concerns regarding food safety and zoonotic transmission risks (Wang et al., 2019; Yang et al., 2024).

Cultural and dietary practices significantly influence the geographical variation in *T. gondii* prevalence across populations (Tenter et al., 2000; Robert-Gangneux and Darde, 2012). Epidemiological investigations consistently demonstrate that consumption of raw or undercooked meat containing viable tissue cysts constitutes a major transmission route, accounting for 30–63% of human infections in high-risk groups such as pregnant women (Kapperud et al., 1996; Belluco et al., 2018; Zhao and Ewald, 2020). Among livestock, sheep, goats, and pigs serve as key intermediate hosts due to their susceptibility and central role in human diets, with national serosurveys in China reporting pooled prevalence rates of approximately 9.9% in goats and 23.2% in pigs (Wei et al., 2021; Yang et al., 2024). Notably, toxoplasmosis is not included in standard ante- or post-mortem meat inspection protocols, allowing infected carcasses to routinely enter the food chain undetected (Dubey et al., 2020a).

The Xinjiang Uygur Autonomous Region represents a critical livestock production hub in northwestern China, contributing approximately 12% of the nation's total mutton output. Statistical communiqué of Xinjiang Uygur Autonomous Region on the 2024 national economic and social development (Xinjiang Uygur Autonomous Region Bureau of Statistics, & NBS Survey Office in Xinjiang, 2025). The region's unique geographical diversity—encompassing the arid Tarim Basin (Southern Xinjiang), the alpine grasslands of the Junggar Basin (Northern Xinjiang), and the agro-pastoral transitional zones of the Turpan-Hami area (Eastern Xinjiang)—creates heterogeneous ecological niches for pathogen transmission (Magalhães et al., 2016). The regional industry is predominantly characterized by extensive pasture-based and semi-pastoral farming systems, where extensive grazing animals frequently interface with environments potentially contaminated by environmentally resistant oocysts shed by definitive hosts. Compounding this risk, local ethnic dietary preferences include the consumption of unpasteurized milk and minimally cooked grilled meats—practices that may inadequately inactivate tissue cysts requiring temperatures above 71 °C (Van den Brom et al., 2020; Nayeri et al., 2020).

Despite Xinjiang's strategic importance in national food security and the environmental plausibility for high transmission risk, systematic epidemiological data on *T. gondii* in local sheep and pig populations remain inadequate. Nevertheless, region-specific infection dynamics, associated risk determinants, and parasitic genetic diversity within Xinjiang's distinct agro-ecological matrices remain inadequately characterized. This critical knowledge deficit substantially impedes the establishment of evidence-based surveillance frameworks and the implementation of targeted intervention strategies.

Therefore, the present study was designed so that these critical gaps would be addressed through the following objectives: (Ahaduzzaman and Hasan, 2022) determination of the current seroprevalence of *T. gondii* in sheep and pigs across Xinjiang's major production areas; (Belaz et al., 2015) identification of key husbandry, environmental, and animal-level risk factors associated with infection; and (Burg et al., 1989) genetic characterization of circulating *T. gondii* isolates by multilocus nested PCR-restriction fragment length polymorphism (Mn-PCR-RFLP).

## 2 Materials and methods

### 2.1 Study area and sample collection

A cross-sectional survey was conducted from April 2024 to July 2025—encompassing primary livestock production cycles—was a cross-sectional survey spanning three ecologically distinct geographical zones of Xinjiang: Northern (Junggar Basin, temperate grassland), Southern (Tarim Basin, arid oasis), and Eastern (Turpan-Hami, agro-pastoral transitional zone). Employed was a multi-stage stratified random sampling approach, with sample sizes calculated to estimate 15% expected prevalence at 95% confidence intervals and 5% precision (*n* = 196 per stratum), adjusted for design effects.

Collected from large-scale intensive farms, smallholder operations, and traditional pastoral systems were blood samples totaling 1011 sheep and 700 pigs. Drawn aseptically via jugular venipuncture (sheep) or cranial vena cava puncture (pigs) into sterile vacuum tubes without anticoagulant were 5–10 mL aliquots of whole blood, transported under cold chain (4 °C) within 6 h and centrifuged at 3000 ×*g* for 10 min. Stored at −20 °C pending serological analysis were the resulting serum aliquots.

Equally critical to food safety assessment were 300 fresh muscle samples (longissimus dorsi, ∼300 mg) from sheep and 300 from pigs, procured independently from municipal abattoirs, wet markets, and supermarkets across identical regions to reflect consumer exposure risk. Maintained at 4 °C during transport and frozen at −20 °C were these specimens, processed for DNA extraction within one week to minimize nucleic acid degradation.

### 2.2 Serological detection by indirect Hemagglutination assay (IHAT)

Serum samples were screened for anti-*T. gondii* antibodies using a commercially IHAT kit (Lanzhou Veterinary Research Institute, China). Serum samples were serially diluted in a 4-fold dilution series from 1:4 to 1:4096. A titer ≥1:64 was considered positive, 1:4–1:16 as suspicious (inconclusive), and < 1:4 as negative, following the manufacturer's protocol and Chinese national veterinary diagnostic standards (GB/T 18448–2020). Each test run included kit-positive and negative controls. (Sun et al., 2020; Wang et al., 2019). The assay was performed strictly in accordance with the manufacturer's protocol.

### 2.3 Molecular detection and genotyping

Genomic DNA was extracted from approximately 300 mg of muscle tissue using a commercial Blood/Cell/Tissue Genomic DNA Extraction Kit (Promega, USA), with final elution in 50 μL of low-EDTA TE buffer (pH 8.0). DNA concentration and purity (A260/A280 ratio) were assessed using a NanoDrop spectrophotometer.

*T. gondii* DNA was detected using semi-nested PCR targeting the B1 gene (131 bp), following established protocols (Burg et al., 1989; Belaz et al., 2015). PCR reactions (25 μL) contained 100–200 ng template DNA, 1× PCR buffer, 1.5 mM MgCl₂, 200 μM dNTPs, 0.4 μM primers, and 1.0 unit Taq polymerase. Positive (*T. gondii* RH strain) and negative (nuclease-free water) controls were included in each run.

B1-positive samples were genotyped using Mn-PCR-RFLP at 10 genetic markers (SAG1, 5’-SAG2, 3’-SAG2, alter. SAG2, SAG3, BTUB, GRA6, c22–8, c29–2, L358, PK1, and Apico) according to Su et al. (2010). Eight reference strains (GT1, PTG, CTG, MAS, TgCgCa1, TgCatBr5, TgCatBr64, TgRsCr1) were included as controls. Digestion products were separated on 2.5–3.0% agarose gels, and banding patterns were compared with ToxoDB database reference profiles.

### 2.4 Statistical analysis

Data were analyzed using SPSS Statistics Version 20.0. Seroprevalence was calculated as the percentage of positive samples out of the total tested. Associations between *T. gondii* seroprevalence and potential risk factors (including region, sex, age group, management mode, and presence of cats) were analyzed using the Chi-square test, with 95% confidence intervals (CIs) reported. Probability (*P*) value < 0.05 was considered statistically significant.

## 3 Results

### 3.1 *T. Gondii* Seroprevalence

The overall *T. gondii* seroprevalence was 11.8% (119/1011; 95% CI: 9.8–13.8) in sheep and 17.6% (123/700; 95% CI: 14.8–20.4) in pigs (Table 1). Regional seroprevalence in sheep was: Southern Xinjiang 10.6% (42/395), Northern Xinjiang 8.6% (18/208), and Eastern Xinjiang 5.3% (14/264). Intensive farming showed lower prevalence (4.9%, 16/329) compared to pasture systems (10.3%, 70/682). In pigs, prevalence was highest in the 2–3 years age group (15.2%, 36/239) compared to 0–1 year (6.3%, 10/155).

**Table 1:** Prevalence of *Toxoplasma gondii* infection in sheep and pig and antibody in Xinjiang Uygur Autonomous Region, China.

Species	No. examined	IHAT titers						Positive		Median	Geometric mean
		1:16	1:64	1:128	1:256	1:1024	1:4096	NO.	%		
Sheep	1011	892	45	14	40	15	5	119	11.8	1:256	1:187
Pig	700	577	58	27	15	14	9	123	17.6	1:128	1:164

**Fig. 1:**
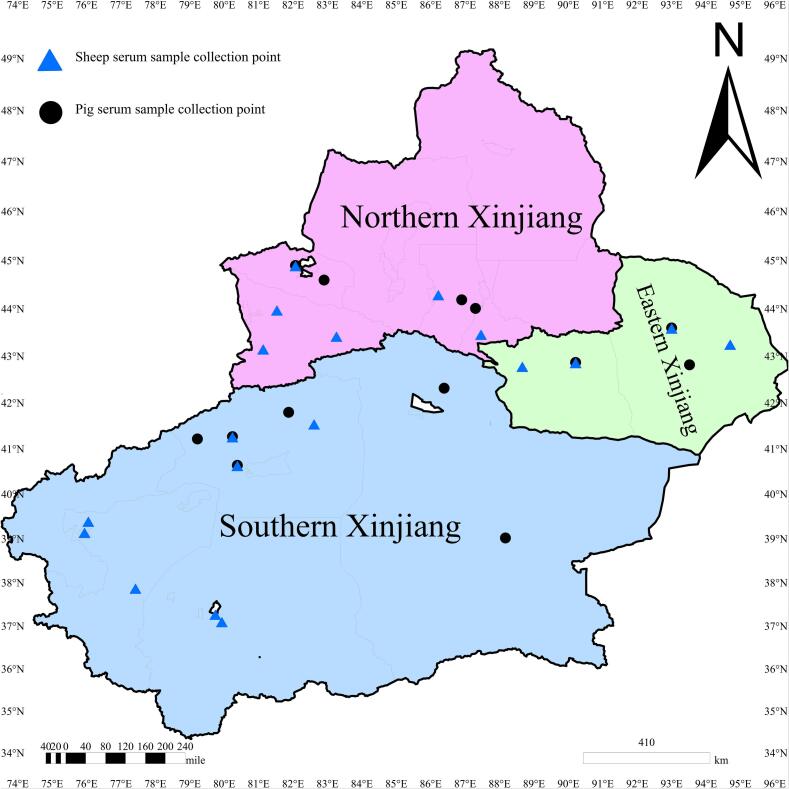
Sample collection sites for sheep and pig serum samples from different regions of Xinjiang Uygur Autonomous Region are marked on the map using ArcGIS 10.8 software (ESRI Inc., Redlands, CA, USA). (http://www.esri.com/software/arcgis/arcgis-for-desktop). The blue triangle represents the collection point of sheep serum samples, and the black circle represents the collection point of pig serum samples. (For interpretation of the references to colour in this figure legend, the reader is referred to the web version of this article.)

Antibody titers in positive sheep ranged from 1:64 to 1:4096 (median: 1:256; Geometric mean 1:187). In pigs, titers ranged from 1:64 to 1:4096 (median: 1:128; Geometric mean 1:164) (See Fig. 1.).

### 3.2 Risk factors analysis

#### 3.2.1 Risk factors for sheep infection with *T. Gondii*

Univariate analysis initially screened five potential risk factors. Further multivariate logistic regression analysis identified four independent risk factors (Table 2). Geographical region was a significant influencing factor; compared to Eastern Xinjiang, the infection risk in sheep from Southern Xinjiang was significantly higher (OR = 2.22, 95% CI: 1.14–3.97, *P* = 0.018). Farming management system showed that the infection risk in extensive grazing sheep was 2.23 times that of intensively reared sheep (95% CI: 1.27–3.90, *P* = 0.005). The presence of cats on farms increased the infection risk by 2.19 times (95% CI: 1.35–3.54, *P* = 0.001). Gender-specific analysis indicated that the infection risk in female sheep was 2.79 times that of males (95% CI: 1.49–5.21, *P* = 0.001). Notably, age was not statistically significant in the multivariate analysis (*P* = 0.742).

**Table 2:** Analysis of risk factors for *Toxoplasma gondi*i infection in sheep in Xinjiang Uygur Autonomous Region, China.

Factor	Category	No. tested	Prevalence (%)	OR (95%CI)	*P*-value
Region	Northern Xinjiang	208	8.6	1.7 (0.82–3.49)	0.158
	Eastern Xinjiang	264	5.3	Reference	
	Southern Xinjiang	395	10.6	2.2 (1.14–3.97)	0.018
Gender	Male	309	3.9	Reference	
	Female	702	10.1	2.79 (1.49–5.21)	0.001
Age	0 < year<1	102	10.8	1.25 (0.61–5.55)	0.546
	1 < year<2	513	9.2	1.04 (0.66–1.65)	0.866
	2 < year<3	396	8.8	Reference	
Presence of free-roaming cat	Yes	493	10.8	2.19 (1.35–3.54)	0.001
	No	518	5.21	Reference	
Management mode	Intensive	329	4.9	Reference	
	Pasture	682	10.3	2.23 (1.27–3.90)	0.005

Bold values indicate statistical significance at the *P* < 0.05 level.

#### 3.2.2 Risk factors for pig infection with *T. Gondii*

Multivariate logistic regression analysis results (Table 3) showed that age was a significant risk factor for *T. gondii* infection in pigs. Compared to that in the 0 < year<1 age group, the infection risk in the 2–3 years age group was significantly higher (OR = 2.58 95% CI: 1.24–5.35, *P* = 0.011). The presence of cats in the farming environment also increased the infection risk by 1.81 times (95% CI: 1.10–3.0, *P* = 0.02). However, regional factors (OR = 1.36, 95% CI: 0.69–2.68, *P* = 0.387) and rodent density on farms (OR = 1.32, 95% CI: 0.51–1.46, *P* = 0.562) did not show statistical significance in this study model.

**Table 3:** Analysis of risk factors for *Toxoplasma gondii* infection in pigs in Xinjiang Uygur Autonomous Region, China.

Factor	Category	No. tested	Prevalence (%)	OR (95%CI)	*P*-value
Region	Northern Xinjiang	285	11.2	1.36 (0.69–2.68)	0.371
	Eastern Xinjiang	153	8.5	Reference	
	Southern Xinjiang	262	12.2	1.44 (0.73–2.83)	0.295
Presence of free-roaming cat	Yes	388	15.5	1.81 (1.10–3.0)	0.02
	No	262	9.2	Reference	
Presence of rodents	Yes	306	8.2	0.86 (0.51–1.46)	0.573
	No	394	9.4	Reference	
Age	0 < year<1	155	6.3	Reference	
	1 < year<2	306	10.2	1.64 (0.78–3.43)	0.193
	2 < year<3	239	15.2	2.58 (1.24–5.35)	0.011

Bold values indicate statistical significance at the *P* < 0.05 level.

### 3.3 Molecular detection and genotyping

Among the sheep muscle samples, 26 (8.7%) were tested positive for *T. gondii* DNA, and 40 (13.3%) pig muscle samples were positive. PCR-positive samples were verified by agarose gel electrophoresis, all showing specific bands of the expected size.

**Fig. 2:**
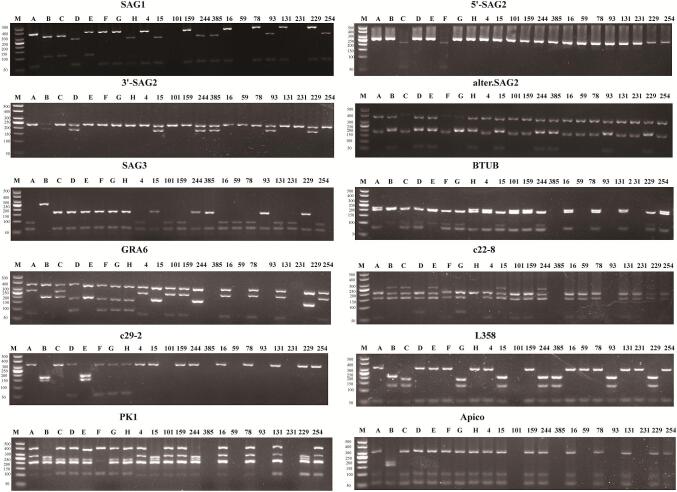
PCR-RFLP results of Toxoplasma gondii isolates from sheep and pig at 12 loci. M: DL500 marker, A-H: Reference strains GT1, PTG, CTG, MAS, TgCgCa1, TgCatBr5, TgCatBr64, TgToucan and TgRsCr1, 4,15,101,159,244,385,16,59,78,93,131,231,229,254: Positive samples.

Genotyping using Mn-PCR-RFLP at ten genetic markers was performed on the PCR-positive samples. Among the 26 sheep-derived isolates, 6 were successfully genotyped, with 4 identified as ToxoDB#9 or ToxoDB#10 (isolates 4, 15, 159, 244), one as potential Type I/ToxoDB#27/35 (isolate 101), and one as ToxoDB#9/20 (isolate 385). Of the 40 pig-derived isolates, 8 were successfully genotyped, with 6 identified as ToxoDB#9 or #10 (isolates 16, 78, 131, 229, 254, and potentially 93), and two as Type I/ToxoDB#24 (isolates 59 and 231) (Fig. 2). The remaining samples could not be assigned a definitive genotype due to incomplete typing signals (Table 4).

**Table 4:** Genotyping of *Toxoplasma gondii* isolates in sheep and pigs in Xinjiang Uygur Autonomous Region, Chi.

Isolate ID	SAGI	5′ + 3′ SAG2	Alt. SAG2	SAG3	BTUB	GRA6	c22–8	c29–2	L358	PK1	Apico	Genotype
GT1	I	I	I	I	I	I	I	I	I	I	I	Reference, Type I,ToxoDB#10
PTG	II/III	II	II	II	II	II	II	II	II	II	II	Reference, Type II, ToxoDB#1
CTG	II/III	III	III	III	III	III	III	III	III	III	III	Reference, Type III, ToxoDB#2
MAS	u-1	I	II	III	III	III	u-1	I	I	I	I	Reference, ToxoDB#17
TgCgCa1	I	II	II	III	II	II	II	u-1	I	u-2	I	Reference, ToxoDB#66
TgCatBr5	I	III	III	III	III	III	I	I	I	u-1	I	Reference, ToxoDB#19
TgCatBr64	I	I	u-1	III	III	III	u-1	I	III	III	I	Reference, ToxoDB#111
TgRsCr1	u-1	I	II	III	I	III	u-2	I	I	I	I	Reference, ToxoDB#52
4 (sheep)	I	I	I	I	I	I	I	I	I	I	I	ToxoDB#10
15 (sheep)	u-1	II	II	III	III	II	II	III	II	II	I	ToxoDB#9
101 (sheep)	N	I	I	I	I	I	I	N	N	I	N	Type I or ToxoDB#27 or ToxoDB#35
159 (sheep)	I	I	I	I	I	I	I	I	I	I	I	ToxoDB#10
244 (sheep)	u-1	II	II	III	III	II	II	III	II	II	I	ToxoDB#9
385 (sheep)	u-1	II	II	III	N	N	N	N	II	N	N	ToxoDB#9 or ToxoDB#20
16 (pig)	I	I	I	I	I	I	I	I	I	I	I	ToxoDB#10
59 (pig)	N	I	I	I	N	N	I	N	I	N	N	Type I or ToxoDB#24
78 (pig)	I	I	I	I	I	I	I	I	I	I	I	ToxoDB#10
93 (pig)	u-1	II	II	III	N	N	N	N	II	N	N	ToxoDB#9 or ToxoDB#20
131 (pig)	I	I	I	I	I	I	I	I	I	I	I	ToxoDB#10
231 (pig)	N	I	I	I	N	N	I	N	I	N	N	Type I or ToxoDB#24
229 (pig)	u-1	II	II	III	III	II	II	III	II	II	I	ToxoDB#9
254 (pig)	I	I	I	I	I	I	I	I	I	I	I	ToxoDB#10

## 4 Discussion

This study comprehensively revealed the epidemiological characteristics of *T. gondii* infection in sheep and pigs in Xinjiang through systematic serological surveys, molecular detection, and genotyping. To our knowledge, this represents one of the most extensive investigations of toxoplasmosis in livestock within this region, providing both serological and molecular evidence for infection status.

The seroprevalence of *T. gondii* in sheep (11.8%) and in pigs (17.6%) in Xinjiang was generally consistent with reports from other regions in China but significantly lower than levels reported in some developed countries.The global pooled seroprevalence in sheep is estimated at 33.86%; while our observed rate is below this average, it falls within the wide reported range (6.6%–44%) observed across diverse geographical regions (Dubey et al., 2020a; Ahaduzzaman and Hasan, 2022). Regional variations likely result from a complex interplay of factors, including local climate, geographical characteristics, farming management practices, and variations in the sensitivity of diagnostic methods (Symeonidou et al., 2023). Within China, the *T. gondii* seroprevalence in sheep in Xinjiang is lower than that reported from Jinzhou, Henan, Zhejiang, and Jiangsu (Xu et al., 2015; Yang et al., 2017), suggesting potential north-south gradients or management practice differences requiring further investigation.

Beyond determining prevalence, our analysis identified critical risk factors shaping local transmission dynamics. Geographically, the significantly higher infection risk observed in sheep from Southern Xinjiang underscores the influence of region-specific environmental or management conditions. Regarding farming systems, the higher *T. gondii* prevalence in extensive grazing sheep compared to those under intensive systems (OR = 2.23) highlights the inherent risk associated with pastoral systems, where animals are more likely to encounter contaminated pastures and wildlife (Dubey et al., 2020b). This finding suggests that promoting improved farming management practices and biosecurity measures in pastoral areas could be effective to reduce environmental contamination and control *T. gondii* transmission.

The strong association between *T. gondii* seroprevalence and the presence of cats is a notable finding, indicating that definitive hosts play an important role in the local epidemiology. Cats shed environmentally resistant oocysts, and their proximity to livestock substantially increases infection risk, aligning with observations from previous studies (Sadiq et al., 2023; Stelzer et al., 2019; Dámek et al., 2023). Additionally, gender-specific analysis revealed that female sheep carried significantly higher infection risk than males (OR = 2.79), potentially related to physiological state changes during the reproductive period, such as immune suppression during pregnancy, or differential husbandry practices providing more exposure opportunities.

Notably, unlike the commonly assumed cumulative exposure risk model (Dubey et al., 2020a), age showed no significant correlation with *T. gondii* infection risk in this study. This phenomenon might indicate the presence of persistent, high levels of oocyst contamination in the Xinjiang environment, leading to similar exposure risks for animals of all age groups—a unique transmission dynamic in the region worthy of further investigation.

Molecular detection results showed that the *T. gondii* DNA detection rate in pigs (13.3%) was higher than in sheep (8.7%), consistent with the serological survey results and further confirming the higher susceptibility of pigs to *T. gondii*. Genotyping studies revealed that ToxoDB#9 and ToxoDB#10 are the dominant genotypes in Xinjiang, consistent with reports from other regions in China including Yunnan (goats; Miao et al., 2015), Zhejiang (pigs; Sun et al., 2022), and Xinjiang horses (Ren et al., 2019), confirming the dominant status of these two genotypes in China's *T. gondii* population (Shwab et al., 2014; Dong et al., 2018; Zhou et al., 2009).

From a public health perspective, these findings have important implications. Residents in Xinjiang have unique dietary practices including consumption of unpasteurized sheep's milk and preference for undercooked meat preparations. *T. gondii* has been successfully detected in unpasteurized sheep's milk, which poses a potential risk (Jiang et al., 2020; van den Brom et al., 2020). Survey data indicated that over 30% of respondents preferred ‘tender grilling’ when cooking lamb skewers—a method that might not heat the internal part of the meat to temperatures sufficient to kill tissue cysts (typically requiring above 71 °C). Traditional dried meat processing techniques might similarly fail to completely inactivate tissue cysts. Therefore, the consumption of undercooked meat containing *T. gondii* tissue cysts may present a health risk to consumers.

Based on these findings, we recommend the following control measures: (i) strengthen the management of cats on farms and implement strict biosecurity measures; (ii) improve farming practices in pastoral areas to reduce environmental contamination; and (iii) enhance public health education to promote thorough cooking of meat before consumption and avoid consumption of unpasteurized dairy products.

Limitations of this study include the inability to complete genotyping for some samples due to poor DNA quality, and the risk factor analysis not covering all potential influencing factors. Critically, this study lacked individual-level pairing between serological and molecular data, as serum samples were collected from live animals on farms while muscle samples were procured from retail channels. Consequently, the presence of tissue cysts in retail meat does not necessarily correlate with seropositivity in the same individual, precluding the calculation of the predictive value of serology for tissue cyst presence. Future research should employ matched sampling designs, expand sampling size, employ more sensitive molecular typing techniques, and conduct long-term longitudinal studies to better understand the transmission dynamics of *T. gondii* in Xinjiang.

## 5 Conclusions

This study revealed a moderate prevalence of *T. gondii* infection in sheep (11.8%) and pigs (17.6%) in Xinjiang, with significant regional and management-related variations. The identification of key risk factors—particularly extensive grazing farming systems (OR = 2.23) and feline presence—provides a basis for developing targeted control strategies. Furthermore, the detection of the predominant genotypes ToxoDB#9 and ToxoDB#10 in mutton and pork underscores the potential for foodborne zoonotic transmission in the region. Based on these findings, public health efforts should focus on: (Ahaduzzaman and Hasan, 2022) enhancing consumer education regarding the risks associated with consumption of unpasteurized dairy products and undercooked meat, and (Belaz et al., 2015) reducing contact between felids and livestock by strengthening on-farm biosecurity measures.

## Ethics approval and consent to participate

The animal study was reviewed and approved by the Ethics Committee of Xinjiang Medical University (No. IACUC-20251001-03).

## CRediT authorship contribution statement

**Wen-Ge Liu:** Writing – original draft. **Jia Huang:** Writing – review & editing. **Maihemudaimu Maihemuti:** Investigation. **Wang Fu:** Software. **Hang-Yu Meng:** Software. **Xiao-Pei Xu:** Supervision, Writing – review & editing.

## Declaration of competing interest

The authors declare that they have no competing interests.
